# Analyzing the Local Electronic Structure of Co_3_O_4_ Using 2p3d Resonant Inelastic X-ray Scattering

**DOI:** 10.1021/acs.jpcc.2c01521

**Published:** 2022-05-11

**Authors:** Ru-Pan Wang, Meng-Jie Huang, Atsushi Hariki, Jun Okamoto, Hsiao-Yu Huang, Amol Singh, Di-Jing Huang, Peter Nagel, Stefan Schuppler, Ties Haarman, Boyang Liu, Frank M. F. de Groot

**Affiliations:** † Debye Institute for Nanomaterials Science, Utrecht University, Universiteitsweg 99, 3584 CG Utrecht, The Netherlands; ‡ Department of Physics, University of Hamburg, Luruper Chaussee 149, G610, 22761 Hamburg, Germany; § Karlsruhe Institute of Technology, Hermann-von-Helmholtz-Platz 1, D-76021 Karlsruhe, Germany; ∥ Deutsches Elektronen-Synchrotron DESY, Notkestraße 85, 22607 Hamburg, Germany; ⊥ Department of Physics and Electronics, Graduate School of Engineering, Osaka Prefecture University 1-1 Gakuen-cho, Nakaku, Sakai, Osaka 599-8531, Japan; # National Synchrotron Radiation Research Center, No. 101 Hsin-Ann Road, Hsinchu Science Park, Hsinchu 30076, Taiwan

## Abstract

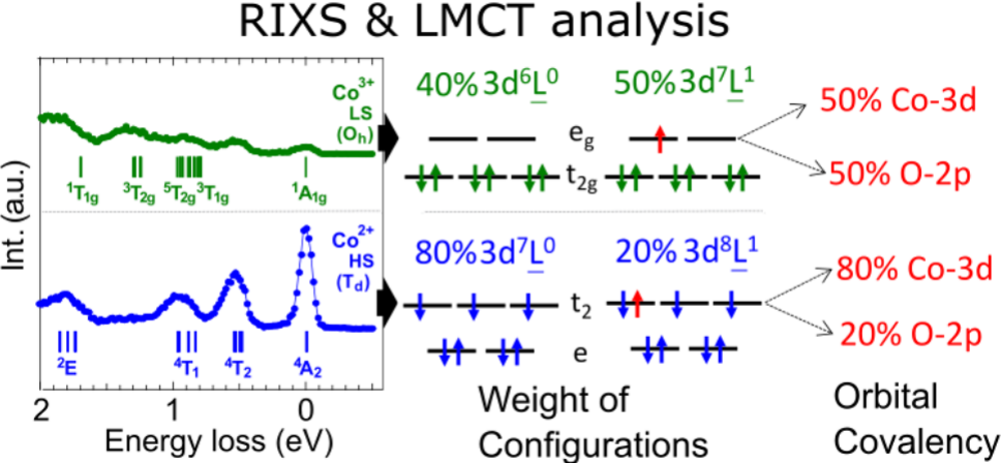

We present the cobalt 2p3d resonant inelastic X-ray scattering (RIXS) spectra of Co_3_O_4_. Guided by multiplet simulation, the excited states at 0.5 and 1.3 eV can be identified as the ^4^*T*_2_ excited state of the tetrahedral Co^2+^ and the ^3^*T*_2*g*_ excited state of the octahedral Co^3+^, respectively. The ground states of Co^2+^ and Co^3+^ sites are determined to be high-spin ^4^*A*_2_(*T*_*d*_) and low-spin ^1^*A*_1*g*_(*O_h_*), respectively. It indicates that the high-spin Co^2+^ is the magnetically active site in Co_3_O_4_. Additionally, the ligand-to-metal charge transfer analysis shows strong orbital hybridization between the cobalt and oxygen ions at the Co^3+^ site, while the hybridization is weak at the Co^2+^ site.

## 1 Introduction

Electronic properties of 3d transition-metal (TM) oxides are governed by the interactions between the TM ions and the neighboring contributions. Those interactions are determined by Hund’s coupling, crystal-field splitting, spin–orbit coupling, and the interatomic hybridization (covalent bonding). The determination of the local electronic structure is a relatively straightforward task for localized materials, but it faces an experimental challenge for a mixed-valence TM compound, especially when the constituting TM ions have a rich overlapping multiplet structure, which is the case for Co_3_O_4_.

Co_3_O_4_ crystallizes in a normal spinel structure (AB_2_O_4_) below 850 K.^1,2^ The Co ions in the A sites with tetrahedral (*T*_*d*_) symmetry are divalent (Co^2+^), while those in the B sites with octahedral (*O_h_*) symmetry are trivalent (Co^3+^) with a small trigonal distortion. An experimental determination of the electronic structure of two inequivalent Co sites is of fundamental importance to elucidate the magnetic coupling responsible for the antiferromagnetic order (below ∼40 K).^3,4^ The Co-site dependence of covalency is crucial for large exchange anisotropies achieved by a substitution of Ni or Fe for Co ions and its application to magneto-optical information storage.^5−7^ The covalency also influences the charge capacity. Co_3_O_4_ shows a high charge capacity (700 mAh/g) and a good cycle performance of nanosize negative electrodes, which makes Co_3_O_4_ an interesting compound for the lithium battery applications.^8,9^ Moreover, the role of Co^2+^ ions in the oxygen evolution reaction step of water oxidation is debated.^10−17^ A small band gap was found in Co_3_O_4_ that is important for the application in a photovoltaic cell,^18−20^ where the electronic structures of individual sites potentially plays an important role.

The electronic structure of Co_3_O_4_ has been characterized by the ultraviolet/visible (UV/vis)^21−25^ and near-infrared (NIR) absorption.^4,26^ These absorption spectra show excitations at 0.82, 0.93, 1.64, and 2.81 eV,^22^ while diverse interpretations for these excitations are proposed,^4,10,21−25^ partly related to the fact that the dd excitations are usually dipole forbidden.^27^ Hibberd et al. revealed distinct absorption peaks for the Co valence (2+ or 3+) and local environment (*O_h_* or *T*_*d*_) of Co_3_O_4_ using Co L_2,3_-edge (2p) X-ray absorption spectroscopy (XAS).^28^ However, due to possible overlap of the multiple Co-site signals and the lifetime broadening (∼200 meV), the details of the local electronic structure are limited.

In this work, we study the Co-site resolved low-energy local excitations in Co_3_O_4_ using Co 2p3d resonant inelastic X-ray scattering (RIXS). By setting the resonant photon energy to the distinct Co L_3_-XAS features of the Co^2+^ and Co^3+^ sites,^28^ the site-selective local excitations are measured. The site-selectivity of RIXS has been discussed in literature.^29−31^ The (photon-in–photon-out) transitions in RIXS brings a broad sensitivity to dd excitations of the system that provides a better determination of the local electronic structure including the orbital hybridization effects.^32−36^ This has also been demonstrated using 1s3d RIXS on Co_3_O_4_,^37,38^ but the intensity was limited by the absorption cross-section of the quadrupole 1s3d transition. We show that the Co 2p3d RIXS is a good chemical site-selective approach to study accurate details of the ground state and the core excited states.

## 2 Methodology

### 2.1 Experimental Details and Sample Preparation

The experiments were performed on 99.9985% Co_3_O_4_ powder produced by Alfa Aesar. A 10 mm × 0.5 mm (diameter × height) cylindrical pellet Co_3_O_4_ was prepared for the measurements. Co 2p XAS spectra were acquired at the soft X-ray WERA beamline of the Karlsruhe Research Accelerator (KARA) synchrotron in Germany. The instrumental resolution was calibrated to be ∼280 meV full width at half-maximum (fwhm) at the Co L_2,3_ edge (∼780 eV). Both the total electron yield (TEY) and the fluorescence yield (FY) methods were employed. The Co 2p3d RIXS measurements with linearly vertical (V) and horizontal (H) polarized incident X-rays were performed at the Taiwan Light Source (TLS) beamline 05A of National Synchrotron Radiation Research Center (NSRRC) in Taiwan.^39^ The experimental energy resolution of the incident photon was ∼700 meV and the combined resolution of RIXS was ∼90 meV. A grazing incident geometry (∼20°) with the spectrometer at 90° was used. Given the energy-compensation principle, the RIXS resolution is much better than the incident photon resolution.^39,40^ For a perfect monochromator-spectrometer pair, the photons will be focused at the mirror position of the photon source. As soon as the photon lost its energy in the path, the focus will not be kept at the same position. Thus, using a position sensitive detector combined with the active monochromator-spectrometer system, the RIXS resolution can be decoupled from the incident photon resolution. To compare the XAS spectra measured at different facilities, the background signals were subtracted from the spectra, as described in the Supporting Information (SI). The subtracted spectra were normalized to the maximum of the Co L_3_ edge. We calibrated the photon energy of the RIXS to the absorption spectra acquired at the WERA beamline. The RIXS spectra measured with horizontal (H) polarization were normalized to the exposure time. Then the spectra with vertical (V) polarization were normalized to the H-polarization spectra according to the profile at high energies (above 2.5 eV). The measurements at the KARA-WERA and TLS-05A beamlines were carried out at 50 and 150 K, respectively. We note that the simulated intensity is given as absolutely cross-section, while the experimental intensity is given in normalized units.

### 2.2 Theory

The results were analyzed using a cluster model that includes the Coulomb multiplet interaction, the crystal-field splitting, the spin–orbit coupling, and the charge transfer between Co 3d and O 2p orbitals. The calculation was carried out with the program Quanty, which implements a configuration-interaction scheme.^41,42^ To study the mixed-valence spectra of Co_3_O_4_, the Co^2+^(*T*_*d*_) site and the Co^3+^(*O_h_*) site were calculated separately. We took the initial parameters from literature for a high-spin Co^2+^ ground state (^4^*B*_1_ in *D*_2*d*_ symmetry) and a low-spin Co^3+^ ground state (^1^*A*_1*g*_ in *O_h_* symmetry).^43−45^ The values were refined by a detailed comparison with the high-resolution RIXS data and its polarization dependence (section 3.2). To evaluate the used values, we compared the ones estimated by an ab initio calculation for Co_3_O_4_. The ab initio calculation was based on a density-functional calculation with the local-density approximation (LDA). The parameters (crystal field and hopping parameters) were extracted from the tight-binding model which spans the Co 3d and O 2p bands by a Wannier projection.

## 3 Results

### 3.1 2p3d RIXS Experimental Results

**Figure 1:**
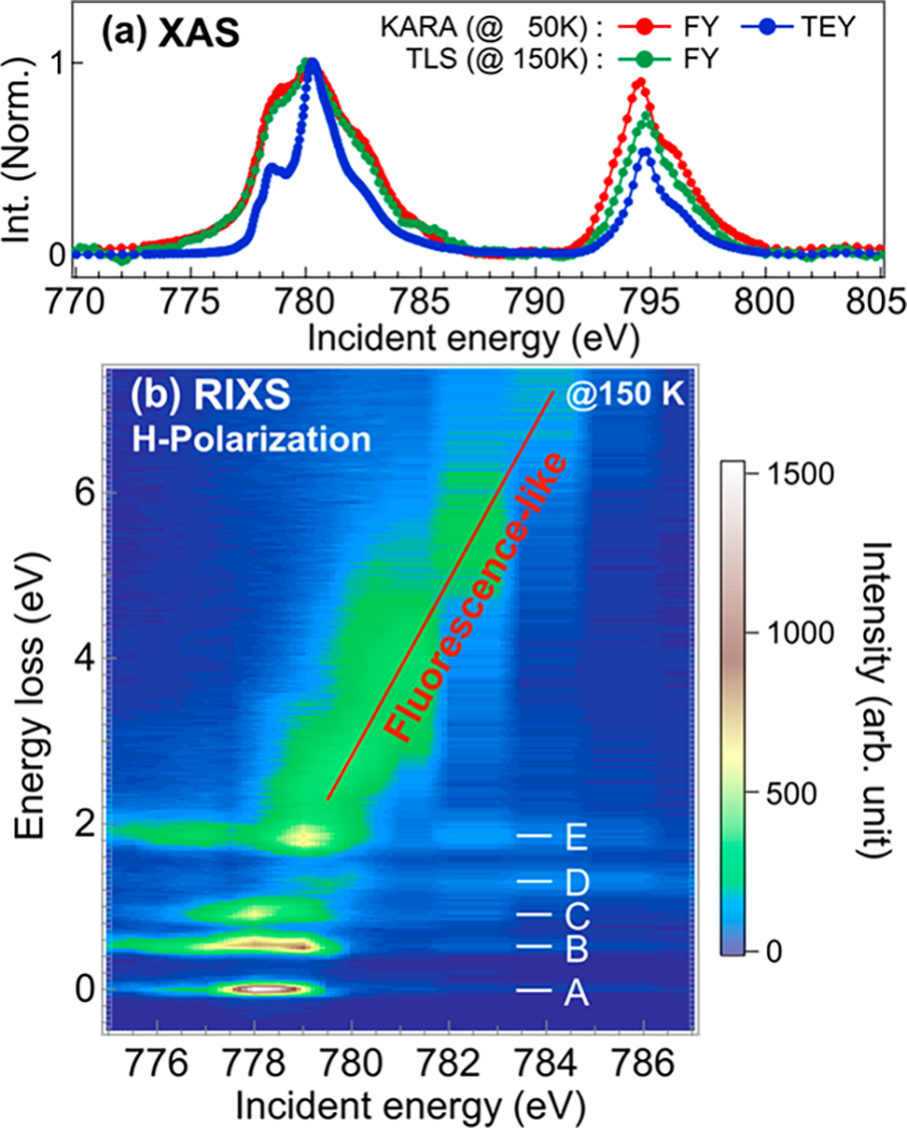
Experimental results. (a) The H-polarization 2p XAS spectra of Co_3_O_4_. (b) The H-polarization 2p3d RIXS energy map of Co_3_O_4_.

The experimental Co 2p XAS and 2p3d RIXS results are presented in Figure 1. The TEY spectrum shows sharp features at 778.5 and 780.2 eV, which are the characteristic signals of the Co^2+^ and the Co^3+^ sites in Co_3_O_4_, respectively.^28^ The FY spectrum, on the other hand, is rather broad, and the features are obscure. This is due to strong saturation and self-absorption effects for a bulk sample. In the 2p3d RIXS result (Figure 1b), a fluorescence-like signal increases with incident photon energies as guided by a red line. Sharp features at ∼0.0, 0.5, 0.9, 1.2, and 1.9 eV are observed and labeled from A to E, respectively. The features A–C are resonantly enhanced at 778 eV, while feature D is enhanced at 780 eV. Feature E resonates at 778.7 eV. These features are attributed to local excitations of the Co^2+^ and Co^3+^ sites, as we will discuss later.

**Figure 2:**
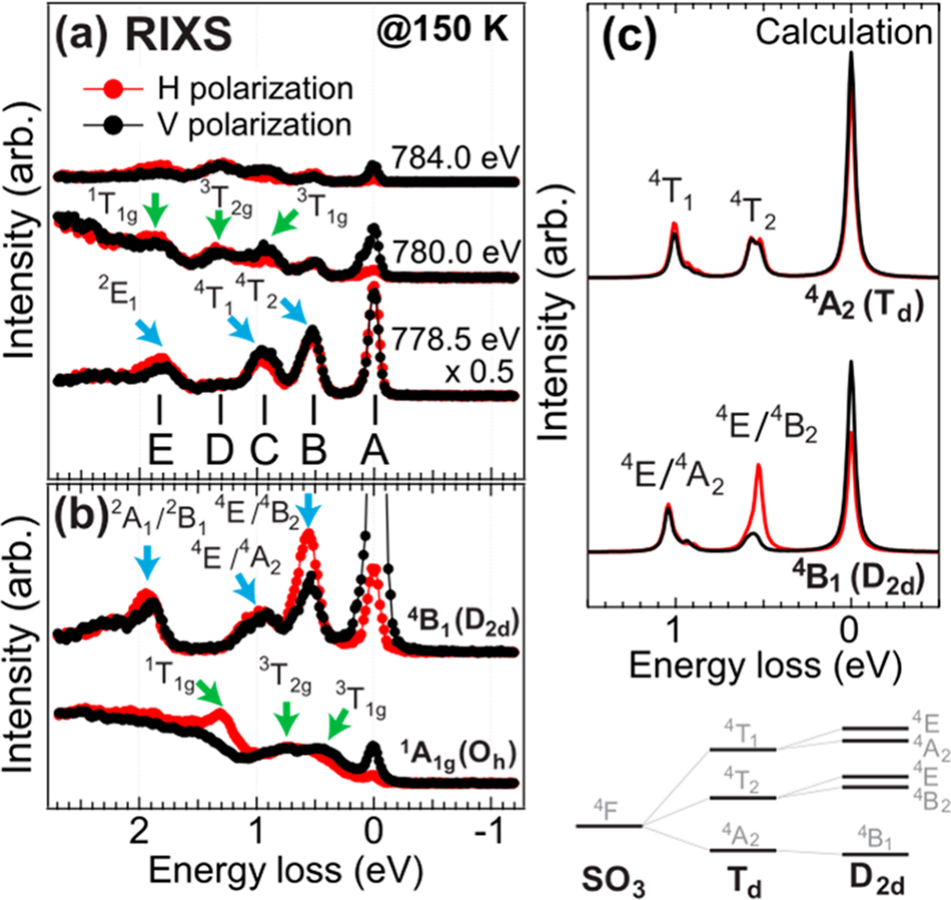
Comparison of H- and V-polarization RIXS spectra of (a) the Co_3_O_4_ and (b) the ^4^*B*_1_(*D*_2*d*_) and ^1^*A*_1*g*_(*O_h_*) ground states from refs (44 and 45). The blue (green) arrows indicate the characteristic features of the Co^2+^(Co^3+^) site. (c) The calculated 2p3d RIXS polarization comparison of distorted and nondistorted tetrahedral Co^2+^ using parameters in ref (44) without considering the ligand-to-model charge transfer.

To gain more insight, Figure 2a compares the H- and V-polarization energy loss spectra at three different incident energies. At 780.0 eV, feature C (at ∼0.9 eV) is enhanced by the V-polarization, while feature E (at ∼1.9 eV) shows opposite behavior. This polarization dependence resembles the ^3^*T*_1*g*_ and ^1^*T*_1*g*_ excited states in LaCoO_3_ at 20 K, where the Co^3+^ ions have the ^1^*A*_1*g*_ ground state, see Figure 2a,b. The energy differences indicate that the crystal field splitting varies between the two. Feature B (at ∼0.5 eV) is identified as the ^4^*T*_2_ excited state of the Co^2+^(*T*_*d*_) site.^5^ As a reference, divalent ^4^*B*_1_(*D*_2*d*_ symmetry) Co^2+^ RIXS at the L_3_ edge of K_5_H[CoW_12_O_40_]·*x*H_2_O is shown in Figure 2b,^44^ where the local structure is a distorted tetrahedral Co^2+^. The distortion changes the ground state symmetry from the ^4^*A*_2_(*T_d_*) to the ^4^*B*_1_(*D*_2*d*_) that gives rise to a strong polarization dependence at the 0.5 eV peak in the RIXS spectra. However, the feature B shows no dichroism in Co_3_O_4_, which suggests that the distortion is negligibly small on the Co^2+^ site and supports the ^4^*A*_2_(*T_d_*) symmetry of the ground state in the calculation.

### 3.2 Multiplet Simulation Results

**Figure 3:**
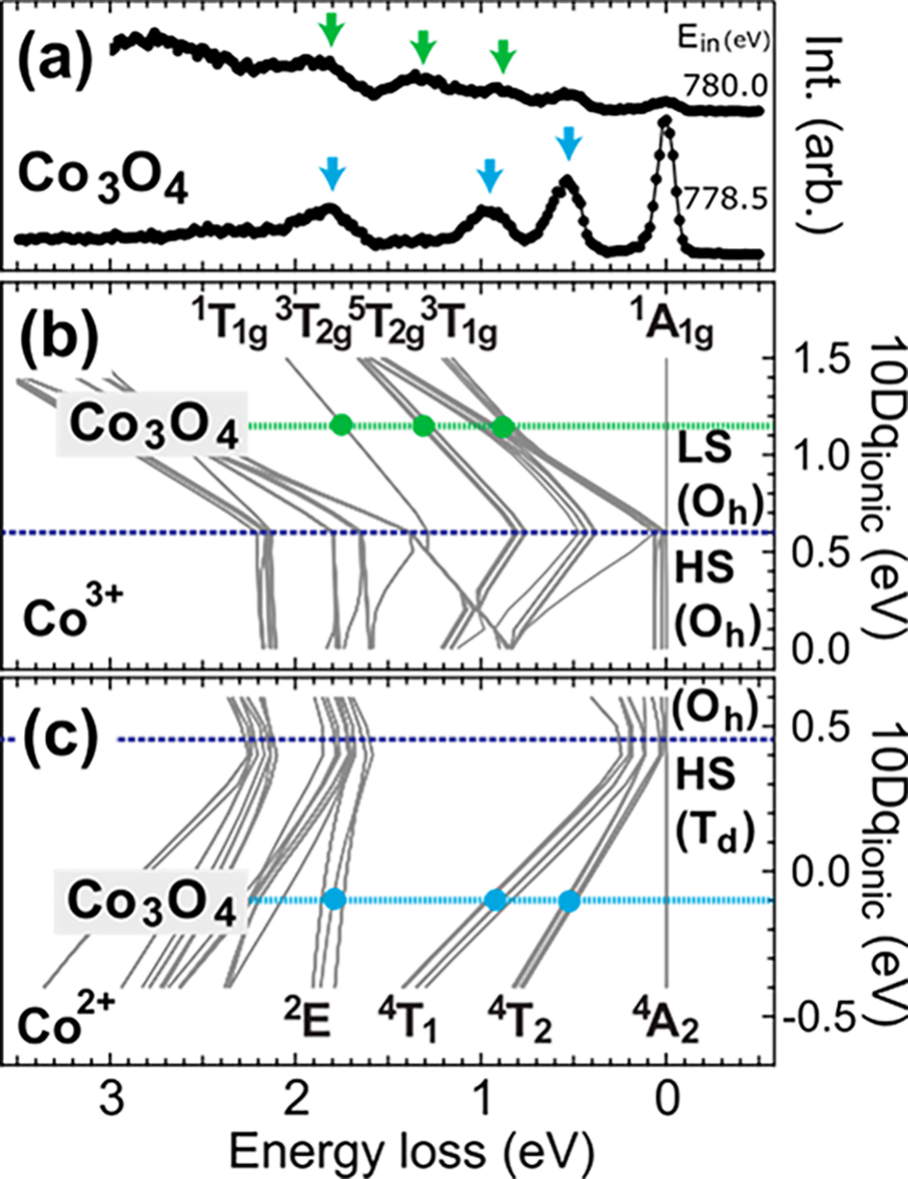
(a) The H-polarization 2p3d RIXS spectra of Co_3_O_4_ excited at 778.5 and 780.0 eV. The calculated energy diagrams of (b) the Co^3+^ ion and (c) the Co^2+^ ion as a function of ionic crystal field energy (10*Dq*_ionic_), including the charge transfer.

Guided by the experimental results, the low-energy local excitations characterize the two Co sites in Co_3_O_4_ and the crystal-field parameters can be determined. In the cluster-model analysis, these parameters identify the symmetry of the local site and the crystal-field energy (10*Dq*) is the key factor to identify the energy splitting of the Co 3d orbitals (t_2g_(t_2_) and e_g_(e) orbitals in *O*_*h*_(*T_d_*) symmetry). For a system involving charge transfer effects, the crystal-field energy can be decomposed into two components:^36^ the ionic crystal field energy (10*Dq*_ionic_) and the effective crystal field energy induced by the charge transfer effect (10*Dq*_CT_). The combination of the two (10*Dq*_tot_) gives the final splitting between the e/e_g_ and t/t_2g_ orbitals. Figure 3b,c shows the excited states energies as a function of the 10*Dq*_ionic_ for the Co^3+^ and Co^2+^ site, respectively. The 2p3d RIXS probed at 780.0 eV shows three features at 0.9, 1.3, and 1.9 eV, indicated with the green arrows in Figure 3a, which are attributed to the signals at the Co^3+^ site. The feature at 0.5 eV shows no polarization dependence, which indicates that the feature is likely the tail contribution of the ^4^*T*_2_ feature of the Co^2+^ site (cf Figure 2). As seen in Figure 3b, the matching of the three features gives 10*Dq*_ionic_ ∼ 1.15 eV at the Co^3+^ site. We note that this value is smaller than the 10*Dq*_tot_ value due to the missing of 10*Dq*_CT_. The 10*Dq*_tot_ values will be given in Table 1. Further discussion about the 10*Dq*_tot_ can be found in the SI. The 10*Dq*_ionic_ value is much larger than the spin-state transition point (10*Dq*_ionic_ ∼ 0.6 eV), suggesting that the ground state on the Co^3+^ site in Co_3_O_4_ is a robust low-spin singlet ^1^*A*_1*g*_. For the Co^2+^ site, the 10*Dq*_ionic_ ∼ −0.1 eV reproduces the energy position of the characteristic features at 0.5, 0.9, and 1.9 eV in the RIXS data probed at 778.5 eV, which are indicated in Figure 3a,c. The negative 10*Dq* value indicates an inversion of the e and t_2_ manifolds in the *T_d_* symmetry.

**Table 1:** Model Parameters Used in the Simulation (in eV), Which Are the Crystal Field Energy, Hopping Integrals, Charge Transfer Energy, *U*_dd_ and *U*_pd_^a^

	10*Dq*_ionic_	10*Dq*_tot_	Δ	*V*_e(e_g_)_	*V*_t_2_(t_2g_)_	*U*_dd_	*U*_pd_
Co_i_^2+^	–0.10	–0.55	4.5	1.0	2.0	4.5	
Co_m_^2+^	–0.02	–0.47	4.5	1.0	2.0	4.5	6.0
Co_i_^3+^	1.15	1.90	1.5	3.12	1.8	6.5	
Co_m_^3+^	0.84	1.59	1.5	3.12	1.8	6.5	7.5

a The i and m stand for the configurations of initial ground state and intermediate state, respectively.

Table 1 summarizes the key parameters, and more values can be found in SI. The charge transfer energy Δ is an energy related to electron transfer from a ligand to the Co site and *V*_e_(g)__/*V*_t_2(g)__ are the values for electron hopping. The *U*_dd_ and *U*_pd_ values parametrize the Coulomb interaction, which are set to reference values.^36,43^ Since the ligand-to-metal charge transfer also contributes to the energy splitting of Co 3d states,^36^ we provide the 10*Dq*_tot_ value that is calculated by the resultant energy splitting by including the charge transfer. To simulate a contraction of Co 3d wave functions by the presence of the core hole,^46^ the 10*Dq*_tot_ value in the intermediate state was reduced from that in the ground state by ∼15%, see also SI.

To evaluate the parameters in Table 1, the values estimated by the LDA calculation are provided. For the Co^2+^(*T*_*d*_) site, the estimated values are −0.10 eV for 10*Dq*_ionic_ and 1.29 (1.82) eV for V_e_ (*V*_t_2__). For the Co^3+^(*O_h_*) site, the estimated values are 0.7 eV for 10*Dq*_ionic_ and 3.03 (1.74) eV for *V*_e_g__ (*V*_t_2g__, where we omitted the energy splitting within the t_2g_ manifolds due to a small trigonal distortion and estimated the *V*_t_2g__ value by averaging the hopping integrals over the two. The *D*σ value, which measures the trigonal distortion, is estimated as 0.05 eV. This value is much smaller than the required value (>0.5 eV) for changing the ground state symmetry (singlet ^1^*A*_1*g*_) of the Co^3+^ site in Co_3_O_4_, but gives a minor correction to the multiplet energies. Because the trigonal distortion gives no visible effect on the spectra, we neglect it in our simulation and the site is referred to as *O_h_* for simplicity. We conclude that the optimized values agree well with the ab initio estimates except for a small discrepancy in the 10*Dq*_ionic_ value at the Co^3+^ site, which is possibly due to an underestimate of the covalency in the LDA scheme.^42,47^

**Figure 4:**
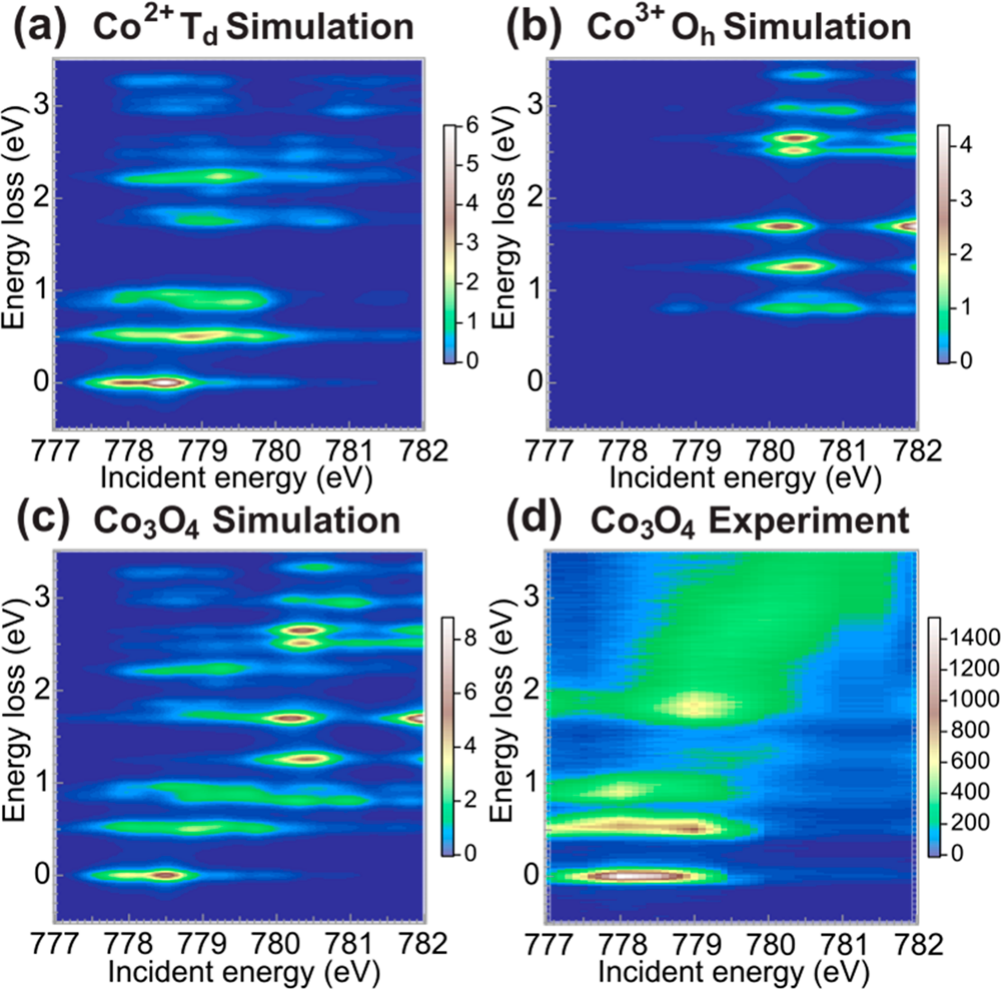
Simulated H-polarization 2p3d RIXS energy maps. (a) Co^2+4^*A*_2_ (*T*_*d*_) ground state. (b) Co^3+1^*A*_1*g*_ (*O_h_*) ground state. (c) The simulation of Co_3_O_4_. (d) Experiment of Co_3_O_4_.

**Figure 5:**
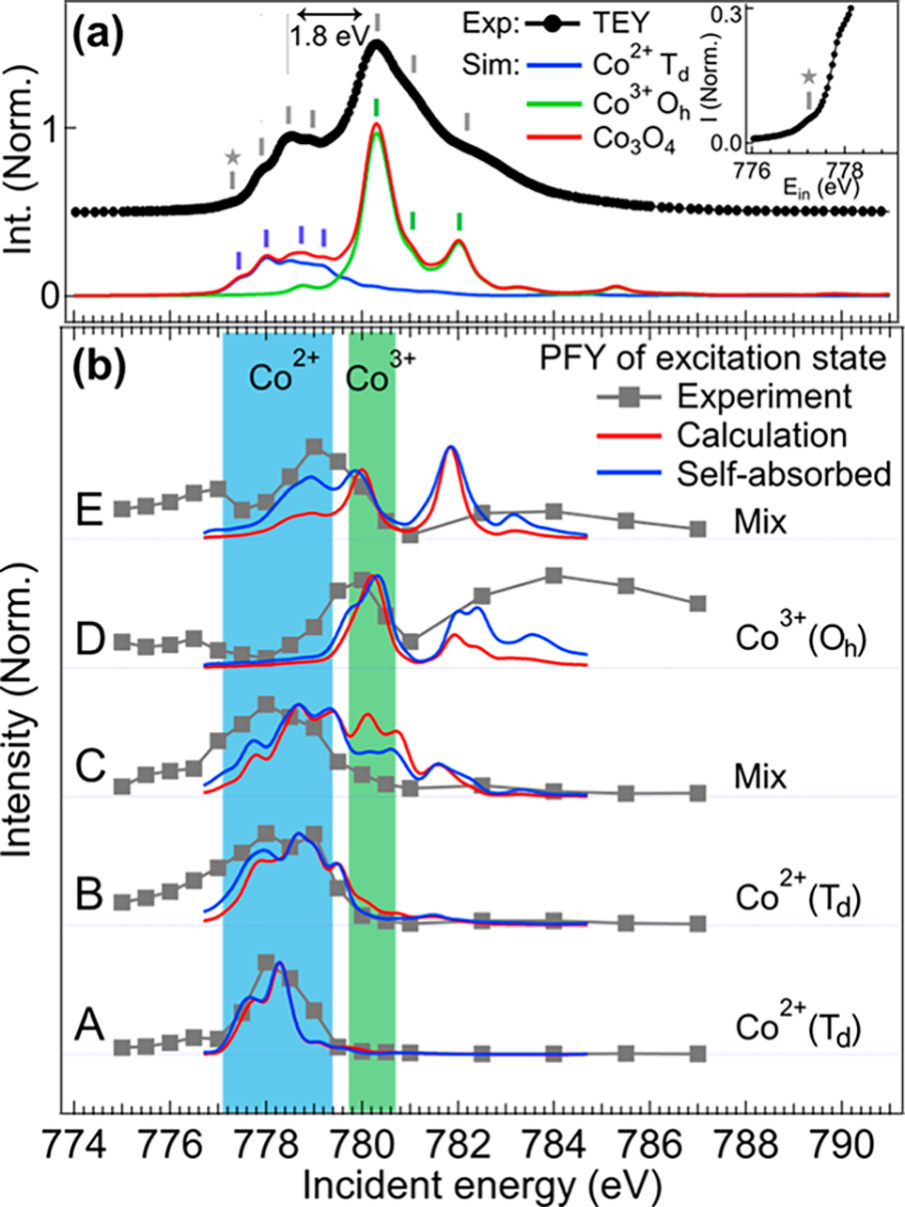
Comparison of XAS spectra. (a) The comparison between the experimental TEY spectrum and the simulations. The inset panel zooms in the pre-edge region and indicates a small feature before the edge. This small feature matches to the first feature indicated in the simulation. (b) The comparison of the PFY spectra correspond to the excited states A–E.

Figure 4a,b shows the RIXS calculation of the Co^2+^ and Co^3+^ sites with the values listed in Table 1. Since the low-spin (^1^*A*_1*g*_) ground state on the Co^3+^ site is angular isotropic, its intensity is strongly suppressed when the scattering angle is about 90° with the H-polarization condition. Thus, the intensity at zero energy loss (elastic line) is mainly due to the Co^2+^ site with the ^4^*A*_2_ ground state. Figure 4c shows the sum of the calculated intensities which was corrected by a combination ratio of stoichiometry (1:2) and the number of holes (3:4), where the ratio was also applied to weight the area normalized XAS spectra. The incident energies of Co^2+^ and Co^3+^ spectra were adjusted by a 1.8 eV difference in between the main L_3_ feature to reproduce the experimental Co L_3_ XAS (cf Figure 5a). This value agrees with the finding in literature (1.7 eV).^28^ The energy shift is not only depending on the oxidation state but also on the spin-state, symmetry (*O_h_*, respectively, *T*_*d*_) and hybridization (charge transfer effect). Some intensity discrepancies are observed, that is, the calculated RIXS at 780.5 eV shows much stronger 1.3, 1.7, and 2.5 eV excited features. These discrepancies can be explained by saturation and self-absorption effects (see section 4.1) and further related to the differential broadening at different site (see section 4.2).

## 4 Discussion

### 4.1 Site Selectivity of Partial Fluorescence Yield Spectra

Partial fluorescence yield (PFY) spectra were acquired for energy transfer of the dd excitations (A–E) from the RIXS map to discuss the local excitations in detail. The experimental data are obtained by fitting the RIXS intensities of these dd excitations (cf Figure 4d), and the theoretical spectra are obtained by summing simulated intensities in an energy window larger than the width of these excitations in Figure 4c. Since the features are well separated in the simulation, this summation represents the peak intensity properly.

Before the discussion on the PFY spectra, the TEY result is compared ahead with the simulated spectra. In Figure 5a, the sum of the simulated XAS of Co^2+^ (blue) and Co^3+^ (green) sites well matches with the experimental TEY data (gray bars and red line). The contributions of two sites are separated in the incident energies that enables to observe site-resolved local excitations by RIXS, as we show in Figure 4. The site selectivity can be better understood from the PFY spectra. In the PFY spectra (Figure 5b), the features A–B and D are unambiguously attributed to the Co^2+^ site and the Co^3+^ site excitations, respectively, while the features C and E show an overlap with both Co^2+^ and Co^3+^ regions. We stress that the overlap is essential since, according to our simulation in Figure 4a,b, both sites have excitations at around 0.9 and 1.9 eV. A systematic discrepancy is observed that the intensity at about 780 eV is always overestimated in the simulation, particularly, of the PFY spectra of the features C and E. To analyze the two features, the saturation and self-absorption effects need to be applied and the correction can be written as^48,49^
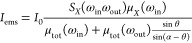
Here, *X*, α, and θ refer to the emission edge of element, the scattering angle, and the sample rotation angle, respectively. In this work, the experimental geometry was set to α = 90° and θ = 20°. The *μ*_tot_(ω_in_) and μ_tot_(ω_out_) are the absorption factor of the photon-in and photon-out channel in the RIXS process. We multiplied a self-absorption coefficient  to the simulated RIXS result (multiplication of *S*_*X*_(ω_in_, ω_out_) and μ_*X*_(ω_in_)). The formula implies that the saturation effect is stronger for a large absorption factor. Thus, the features at ∼780 eV in PFY spectra are to be suppressed. Consequently, the PFY weights on the Co^3+^ region is largely suppressed for the feature C (cf. Figure 5b). It also enhances the intensity at ∼782 eV of PFY spectra for the feature D and shows better agreement with the experiment. However, there is still an intensity discrepancy of the feature at 782 eV for excitation E. This overestimation of 782 eV feature is due to the model of oxygen 2p orbitals, where its bandwidth is neglected. In our simulations, the energy broadening was assumed to be the same for both sites, which yields rather sharp features in the Co^3+^ site contribution compared to the experiment. A larger energy broadening for the Co^3+^ site is caused by the strong ligand–metal hybridization on the Co^3+^ site. To reproduce the broad XAS/PFY structure above the L_3_ edge, the band formation of ligand 2p states must be taken into accounted, which is beyond the description of the cluster model used in this study.

The present analysis shows that the excited states at 0.5 and 1.3 eV are the unique features to identify the ^4^*T*_2_(*T*_*d*_) excited state on the Co^2+^ site and ^3^*T*_1*g*_(*O_h_*) excited state on the Co^3+^ site. In comparison with the infrared^4^ and 1s3d RIXS,^37,38^ those individual site can be resolved much better. Our result confirms that the Co_3_O_4_ is mainly composed by the magnetically active high-spin Co^2+^(*T*_*d*_) and the diamagnetic low-spin Co^3+^(*O_h_*).

### 4.2 Ligand–Metal Hybridization Influence of Different Co Sites in Co_3_O_4_

**Figure 6:**
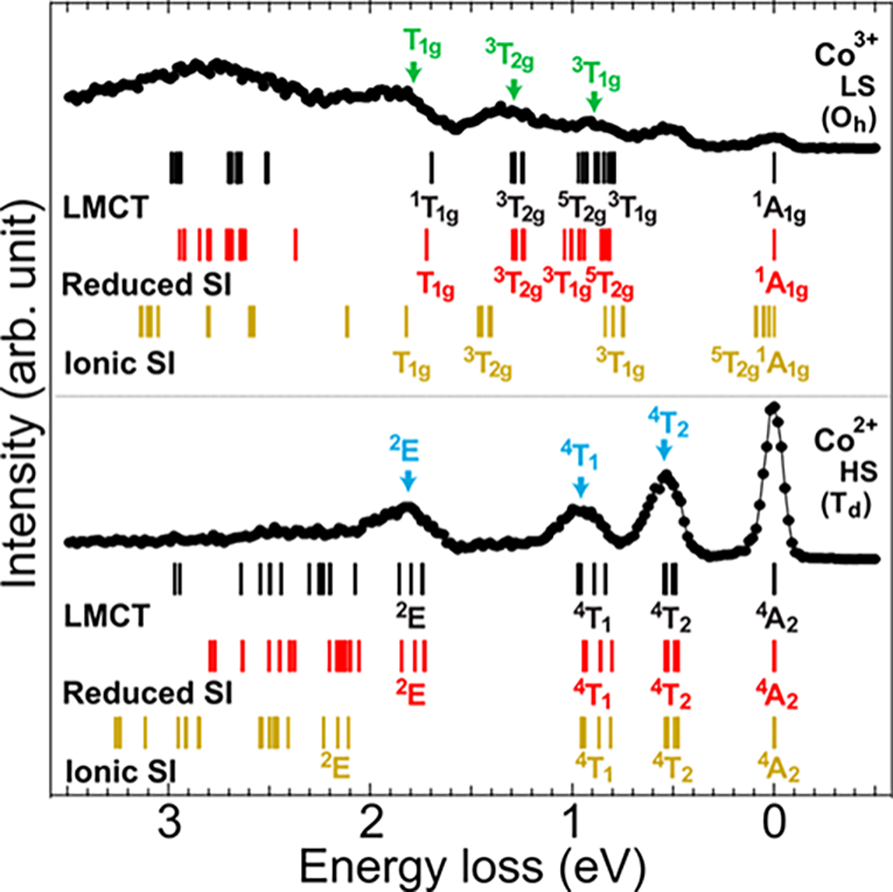
Energy diagrams calculated using the values of the ionic Slater integrals (ionic SI), the reduced Slater integrals (reduced SI), and the ligand-to-metal charge transfer (LMCT) effect.

We discuss here the question whether the ligand–metal hybridization influences the local electronic structure based on the ligand-to-metal charge transfer model. Hibberd et al. have shown in their ionic-model analysis that the Slater integrals of the ionic Coulomb multiplet need to be reduced substantially to reproduce the Co L_3_ XAS spectra, which implies that the ligand–metal hybridization is strong in Co_3_O_4_.^18,28,50^ The sensitivity of 2p3d RIXS to the dd excitations allows us to address the question, and furthermore study the site-dependence of the covalency in Co_3_O_4_. Figure 6 compares the energy diagrams obtained with three different models: (i) the ionic model with the bare (ionic) values of the Slater integrals, (ii) the one with reduced Slater integrals, and (iii) the cluster model including the ligand-to-metal charge transfer channel explicitly. Apparently, the ionic model (i) overestimates energies of observed excitations. For the Co^2+^ site, the ionic model (ii) with reduced Slater integrals (80% from the ionic values) yields good agreement with the experimental data. Note that both model (i) and (ii) show nice agreement on the ^4^*T*_2_ and ^4^*T*_1_ excited states, which indicates that those features are less sensitive to the hybridization change. On the other hand, for the Co^3+^(*O_h_*) site, the Slater integrals are reduced to 55% (80%) from the ionic *F*^2^_dd_ (*F*^4^_dd_) value to fit the experimental data. These unconventional reduction rates for the Co^3+^(*O_h_*) site imply that the differential screening effect via a ligand-to-metal charge transfer channel is not negligible. The cluster model including the ligand-to-metal charge transfer channel shows good agreement to the experimental results.

To obtain further information about the ligand–metal hybridization, Table 2 shows the configuration weights in the ground state of the cluster model using the optimized parameters in Table 1. The ligand-to-metal charge transfer channel mixes the ionic configuration (3d^*n*^) with ones with ligand holes (3d^*n*+1^L^1^ and 3d^*n*+2^L^2^). In the Co^3+^ site, the ligand-hole configurations (3d^7^L^1^ and 3d^8^L^2^) show large weights, which indicates that the Co^3+^ site is strongly hybridized with the ligand states. In contrast, the ligand-hole configuration (3d^8^L^1^) contributes only ∼20% to the ground state in the Co^2+^ site. This observation suggests that the Co^3+^ site is highly covalent, while the Co^2+^ site is rather ionic in Co_3_O_4_. The difference also affects the RIXS profile: the ionic Co^2+^ site shows sharp local dd excitations; the covalent Co^3+^ site exhibits a broad intense fluorescence-like feature (cf. Figure 1).

**Table 2:** Weight of Configurations and Cation Orbital Covalency in the Ground State (unit in %)^a^

	weight of configurations			orbital covalency	
	|3d^*n*^⟩	|3d^*n*+1^L^1^⟩	|3d^*n*+2^L^2^⟩	e(e_g_)	t_2_(t_2g_)
Co^2+^(3d^7^)	79	20	1	100	80
Co^3+^(3d^6^)	40	50	10	50	100

a Although the number of ligand holes is considered up to two in the spectral simulations, the covalency is estimated only using the configurations up to one ligand hole.

The orbital covalency of the Co^2+^ (Co^3+^) cation was analyzed using the approach described in the SI(36) and is summarized in Table 2. The orbital covalencies of e and t_2_ orbitals on the tetrahedral Co^2+^ cation in Co_3_O_4_ are 100% and 80%, respectively. The e orbital is fully occupied and cannot participate in the ligand–metal hybridization, thus 100% orbital covalency is found. A high value of the cation orbital covalency for t_2_ orbital indicates that it less contributes to the ligand-hole configuration d^8^L, which is consistent with the ionic character of the Co^2+^ site. For the Co^3+^ site, the orbital covalencies of e_g_ and t_2g_ orbitals are 50% and 100%. This indicates that the ligand–metal hybridization mainly influences to the e_g_ orbital of the Co^3+^ ions. Although the Co^3+^ cation is the singlet ground state (t_2g_^6^e_g_^0^ state in the ionic picture), the e_g_ orbital forms a strong bonding with neighboring oxygen 2p orbitals. This bonding is represented by the d^7^L configuration in an e_g_ symmetry, yielding the reduced value (50%) of the orbital covalency.

## 5 Conclusions

We present the Co 2p XAS and 2p3d RIXS experimental results in comparison with cluster model simulations on Co_3_O_4_. The 2p3d RIXS provides good chemical site selectivity to the local electronic structure, from which we can identify orbital covalencies of different ions in the compound. The polarization-dependent analysis indicates the symmetry character of the dd excitations, which provides a solid guide to analyze the local electronic structure. By selecting characteristic excitations, the PFY spectra are able to give additional site dependent information. The result shows the ^4^*T*_2_ excited state of the tetrahedral Co^2+^ site at 0.5 eV, which is beyond the discriminative power of optical absorption. In addition, the ^1^*A*_1*g*_ to ^3^*T*_2*g*_ excitation of the octahedral Co^3+^ site at 1.3 eV can be uniquely identified. The ground state electronic structure of the Co^2+^ ions and the Co^3+^ ions are respectively high-spin ^4^*A*_2_(*T*_*d*_) and low-spin ^1^*A*_1*g*_(*O_h_*), where the high-spin Co^2+^ must be the magnetically active site. Our result also shows strong ligand–metal hybridization on the Co^3+^ site, which indicates that the Co^3+^ site in Co_3_O_4_ is rather covalent. In contrast, the Co^2+^ site shows weak hybridization implying that Co^2+^ is more ionic. This chemical site selectivity will help the further understanding on the site-dependent catalytic activity and magnetic activity of the spinel oxides.

## Supporting Information Available

Details about background subtraction of the XAS spectra (section A), an estimation of the theoretical absorption background (section B), all used parameters (section C), the comparison of the simulated XAS spectra (section D), and an explanation of the orbital covalency (section E) (PDF)
