# Supplementary material for: Analyzing the Local Electronic Structure of Co3O4 Using 2p3d Resonant Inelastic X-ray Scattering
Source: J Phys Chem C Nanomater Interfaces. 2022 May 11;126(20):8752–9. doi: 10.1021/acs.jpcc.2c01521 (PMC9150098; doi:10.1021/acs.jpcc.2c01521)
Supplement: Supplementary file 1 — jp2c01521_si_001.pdf [file jp2c01521_si_001.pdf]

Supporting Information of “Analyzing the Local Electronic Structure of Co_3O_4 using 2p3d Resonant Inelastic X-ray Scattering”

Ru-Pan Wang,[†,‡] Meng-Jie Huang,[§,∥] Atsushi Hariki,[⊥] Jun Okamoto,[@] Hsiao-Yu Huang,[@] Amol Singh,[@] Di-Jing Huang,[@] Peter Nagel,[§] Stefan Schuppler,[§] Ties Haarman,[†] Boyang Liu,[†,*] and Frank M. F. de Groot[†,*]

[†]Debye Institute for Nanomaterials Science, Utrecht University, Universiteitsweg 99, 3584 CG Utrecht, The Netherlands

[‡]Department of Physics, University of Hamburg, Luruper Chaussee 149, G610, 22761 Hamburg, Germany

[§]Karlsruhe Institute of Technology, Hermann-von-Helmholtz-Platz 1, D-76021 Karlsruhe, Germany

[∥]Deutsches Elektronen-Synchrotron DESY, Notkestraße 85, 22607 Hamburg

[⊥]Department of Physics and Electronics, Graduate School of Engineering, Osaka Prefecture University 1-1 Gakuen-cho, Nakaku, Sakai, Osaka 599-8531, Japan

[@]National Synchrotron Radiation Research Center, No.101 Hsin-Ann Road, Hsinchu Science Park, Hsinchu 30076, Taiwan

A. 2p XAS spectra background subtraction

Figure S1 shows the raw 2p XAS spectra (red), subtracted 2p XAS spectra (blue), and the background profile (black). We subtracted the background signal from the original XAS results, where the background signal contains edge jump(s), particles scattering, and linear signal. The subtracted spectra were normalized to the maximum of the Co L_3-edge. The photon energy of RIXS beamline were calibrated to the spectra acquired in WERA beamline, where the calibration also applied to the incident energy of RIXS spectra.

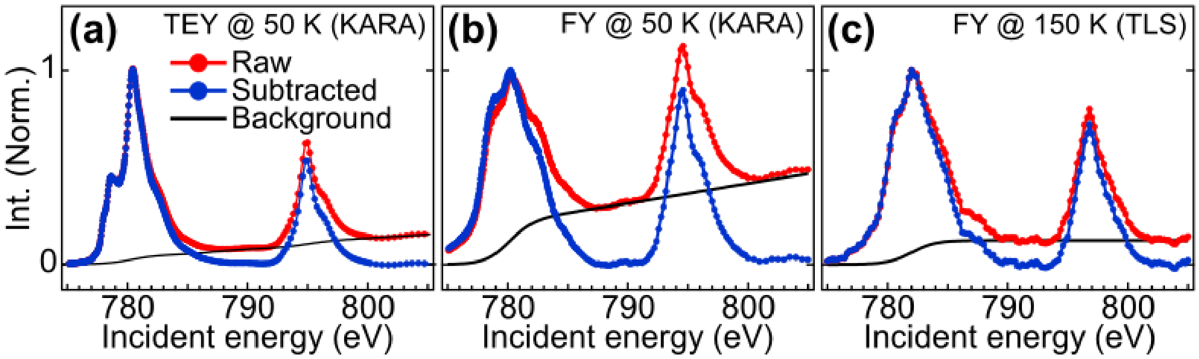

Figure S1: The data treatment of XAS spectra. (a) The TEY-XAS and (b) the FY-XAS spectra measured at WERA beamline in KARA. (c) The FY-XAS spectra measured at soft X-ray RIXS beamline in TLS.

B. Theoretical absorption background estimation

According to the tabulated data [s1], we can estimate the attenuation length for the individual elements of Co_3O_4 (ρ = 6.11 g/cm^3). The partial density of cobalt and oxygen elements (ρ_{Co} and ρ_O) are 4.49 g/cm^3 and 1.62 g/cm^3, respectively. So the attenuation lengths at 780 eV for the cobalt and oxygen elements in the Co_3O_4 are expected to be ~140 nm and ~700 nm, respectively. But the attenuation lengths at the absorption edge are likely overestimated using the Henke's table. For cobalt metal (ρ = 8.9 g/cm^3), the estimated attenuation length is ~75 nm but the experimental results indicate that the attenuation length was ~25 nm at the peak maximum [s2]. Thus, we estimated the value within the range from 25 nm to 140 nm for the attenuation length of cobalt element at 780 eV.

The weighting of μ is proportional to the inverse of the attenuation length that weighting of μ for Co and O are estimated to be 96-83% and 4-17% (attenuation lengths are 25-140 and 700 nm). For pure Co_3O_4, the background absorption (μ_B) at 780 eV is the contribution of the oxygen absorption (the contributions of other edges were omitted). Thus, a value of ~10% of the μ_{max} is suitable estimation for the μ_B.

C. Used parameters and the effective crystal field energy

Table S1-S4 give the used parameters. The Slater integrals F^2_{dd}, F^4_{dd}, F^2_{pd}, G^1_{pd}, and G^3_{pd} as well as the U_{dd} and U_{pd} are used to determine the Coulomb interaction. The Slater integrals were taken to an ionic scheme, where ~80%(75%) of the values from the Hartree-Fock approximation is used for the Co^{2+} (Co^{3+}). The U_{dd} and U_{pd} values were set to the reference values. ζ_p and ζ_d describe the spin-orbit interaction. The charge transfer energy Δ and the hopping integrals $V_{e(e_g)}/V_{t_2(t_{2g})}$ mimic the energy splitting between two configurations and the electron hopping intensity from one configuration to another one.

The crystal field energy $10Dq$ identifies the energy different between the $e(e_g)$ and the $t_2(t_{2g})$ orbitals in the $T_d(O_h)$ symmetry. Once the ligand-to-metal charge transfer is included, the total effective crystal energy $10Dq_{tot}$ is composed by two different parts: (i) ionic crystal field energy of cobalt 3d shell ($10Dq_{ionic}$) and (ii) additional contribution caused by charge transfer and exchange interaction ($10Dq_{CT}$) [s4]. The $10Dq_{tot}$ in currently work can be estimated by $^1A_{1g}$-$^1T_{1g}$ and $^4A_2$-$^4T_2$ excited states energy for the octahedral Co^{3+} and the tetrahedral Co^{2+} sites, which are ~1.90 eV and

~−0.55 eV, respectively. The negative sign on the tetrahedral symmetry infers to the inverse of t_2 and e orbitals with respect to the octahedral symmetry. In contrast, for the simulation considering the charge transfer and exchange interaction effects, the $10Dq_{\text{ionic}}$ should be further reduced to 1.15 eV and −0.1 eV for the octahedral Co^{3+} site and tetrahedral Co^{2+} site, respectively. Our theoretical crystal field energy values (obtained by LDA calculation) considered only the values applied on the Co 3d orbitals, which means the charge transfer induced crystal field energy splitting was not involved. Thus, only $10Dq_{\text{ionic}}$ of cobalt 3d shell has been compared in the main text. We note that the contraction induced by the core hole is applied to the whole valence state wave function (correspond to $10Dq_{\text{tot}}$), thus we applied the $10Dq_{\text{tot}}$ value of the intermediate state is reduced by ~15% in comparison with the ground state [s3] (1.59 eV for the Co^{3+} site and −0.47 for the Co^{2+} site).

In the simulation, a 300 meV (FWHM) Lorentzian convoluting a 300 meV (FWHM) Gaussian was used to simulate the intrinsic broadening and the instrumental broadening of the incident beam. It provides a 0.6 eV total width. For the RIXS spectra, the same incident beam width was applied. In addition to it, a 50 meV (FWHM) Lorentzian convoluting a 60 meV (FWHM) Gaussian was used for the emitted beam, which implies a total width 0.11 eV. These values are comparable to the experimental setting. Nevertheless, we note that the intrinsic broadening was fixed to a value in the current simulation.

Table S1. The values used for Slater integral, spin-orbit coupling energies (in eV). The i and m stand for the configurations of initial ground state and intermediate state, respectively.

	F^2_{dd}	F^4_{dd}	F^2_{pd}	G^1_{pd},	G^3_{pd}	ζ_p	ζ_d
$\mathbf{Co}_i^{2+}$	9.284	5.767	-	-	-	-	0.066
$\mathbf{Co}_m^{2+}$	9.917	6.166	5.808	4.318	2.455	9.748	0.066
$\mathbf{Co}_i^{3+}$	9.371	5.859	-	-	-	-	0.055
$\mathbf{Co}_m^{3+}$	9.932	6.212	5.925	4.463	2.540	9.747	0.055

Table S2. The values used for crystal field energy, charge transfer energy, hopping integrals, U_{dd} and U_{pd} (in eV).

	$\mathbf{10}\boldsymbol{Dq}_{\textbf{ionic}}$	$\mathbf{10}\boldsymbol{Dq}_{\textbf{tot}}$	Δ	$V_{e(e_g)}$	$V_{t_2(t_{2g})}$	U_{dd}	U_{pd}
$\mathbf{Co}_i^{2+}$	−0.10	−0.55	4.5	1.0	2.0	4.5	-
$\mathbf{Co}_m^{2+}$	−0.02	−0.47	4.5	1.0	2.0	4.5	6.0
$\mathbf{Co}_i^{3+}$	1.15	1.90	1.5	3.12	1.8	6.5	-
$\mathbf{Co}_m^{3+}$	0.84	1.59	1.5	3.12	1.8	6.5	7.5

Table S3. The Slater integral, spin-orbit coupling energies values for the case of reduced Slater integral (in eV).

	F^2_{dd}	F^4_{dd}	F^2_{pd}	G^1_{pd},	G^3_{pd}	ζ_p	ζ_d
$\mathbf{Co}_i^{2+}$	7.543	4.686	-	-	-	-	0.066
$\mathbf{Co}_i^{3+}$	5.065	4.750	-	-	-	-	0.055

Table S4. The crystal field energy, charge transfer energy, hopping integrals, U_{dd} and U_{pd} values for the case of reduced Slater integral (in eV).

	$\mathbf{10}\boldsymbol{Dq}_{\textbf{ionic}}$	$\mathbf{10}\boldsymbol{Dq}_{\textbf{tot}}$	Δ	$V_{e(e_g)}$	$V_{t_2(t_{2g})}$	U_{dd}	U_{pd}
$\mathbf{Co}_i^{2+}$	−0.50	-	-	-	-	-	-
$\mathbf{Co}_i^{3+}$	1.95	-	-	-	-	-	-

D. The comparison of the simulated XAS spectra with and without ligand-to-metal charge transfer effect

Figure S2 presents the comparison of the simulated spectra with and without ligand-to-metal charge transfer effect using the parameters in Table S1, S2 (using charge transfer parameters) and Table S3, S4 (using Slater reduction), respectively. Overall, the spectra look similar. The simulation including the ligand-to-metal charge transfer at both Co^{2+} and Co^{3+} sites shows better agreement.

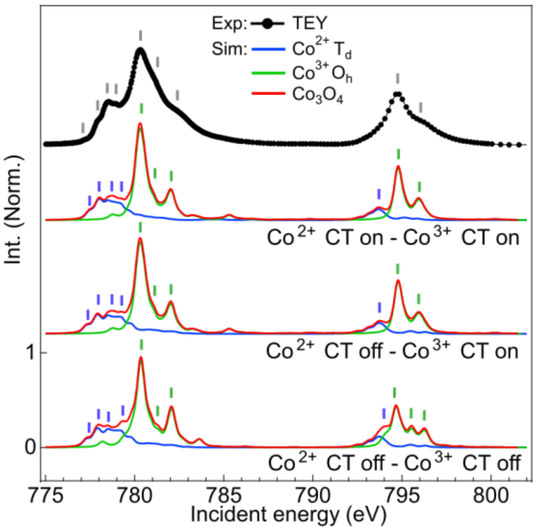

Figure S2: Comparison of the simulated spectra with and without ligand-to-metal charge transfer effect.

E. Estimating the differential orbital covalency of a cation from the cluster model

Including the ligand-to-metal charge transfer effect suggests that the ground state configuration is a mixture of the 3d orbit and ligand hole (L). We calculated the weight of configurations up to two ligand holes and list them in Table S5. Then, we further estimated the cation orbital covalency of Co^{2+} and Co^{3+} cations using the following relation [s4-s6]:

$$Cation\ Orbital\ Covalency(\gamma) = 100\% - N\frac{P_\gamma}{P_{sum}},$$

where γ stands for the state corresponding to the e(e_g) or t_2(t_{2g}) orbitals. 100% indicates to the target orbital which is dominated by the ionic configuration. The coefficient N is a renormalization factor of the number of holes in the orbit out of number of holes in 3dn configuration. For example, in the case of high-spin Co^{2+}(T_d), there are three holes in the t_2 orbit out of three holes in $3d^7$ configuration. Hence the renormalization factor is equal to one ($\frac{\text{number of holes in } 3d^n}{\text{number of holes in } t_2} = 1$). In contrast, the renormalization factor for the e orbit is meaningless because it is fully occupied (no hole exists). The P_γ is the percentages for the configurations which accept the elections transfer from ligand to the orbital. Note here that we only consider one electron transfer in the covalency estimation. P_{sum} is the percentages summation of all possible configurations involved in the hybridization, which is equal to one in this work. Thus, the cation orbital covalency of t_2 orbital on the Co^{2+} site and e_g orbital on Co^{3+} site are given as ~80% and ~50%, respectively.

Table S3. The weight of configurations and orbital covalency in ground state (unit in %). Although the number of ligand holes is considered up to two in the spectral simulations, the covalency is estimated only using the configurations up to one ligand hole.

	$\|3d^n\rangle$	$\|3d^{n+1}\underline{L}^1\rangle$	$\|3d^{n+2}\underline{L}^2\rangle$	e(e_g) covalency	t_2(t_{2g}) covalency
$Co^{2+}(3d^7)$	79	20	1	100	80
$Co^{3+}(3d^6)$	40	50	10	50	100

References

[s1] B. L. Henke, E. M. Gullikson, and J. C. Davis, Atomic Data and Nuclear Data Tables 54, 181 (1993).

[s2] C. T. Chen, Y. U. Idzerda, H.-J. Lin, N. V. Smith, G. Meigs, E. Chaban, G. H. Ho, E. Pellegrin, and F. Sette, Phys. Rev. Lett. 75, 152 (1995).

[s3] S. P. Cramer, F. M. F. de Groot, Y. Ma, C. T. Chen, F. Sette, C. A. Kipke, D. M. Eichhorn, M. K. Chan, W. H. Armstrong, E. Libby et al, J. Am. Chem. Soc. 113, 7937 (1991).

[s4] R.-P. Wang, B. Liu, R. J. Green, M. U. Delgado-Jaime, M. Ghiasi, T. Schmitt, M. M. van Schooneveld, and F. M. F. de Groot, J. Phys. Chem. C 121, 24919 (2017).

[s5] M. U. Delgado-Jaime, J. Zhang, K.; Vura-Weis, and F. M. F. de Groot, J. Synchrotron Rad. 23, 1264 (2016).

[s6] E. C. Wasinger, F. M. F. de Groot, B. Hedman, K. O. Hodgson, and E. I. Solomon, J. Am. Chem. Soc. 125, 12894 (2003).
